# Emergency Physicians Choose Wisely When Ordering Plain Radiographs for Low Back Pain Patients

**DOI:** 10.7759/cureus.3126

**Published:** 2018-08-10

**Authors:** Rashi Hiranandani, Meaghan J Mackenzie, Dongmei Wang, Tak Fung, Eddy Lang

**Affiliations:** 1 Medicine, University of Ottawa, Ottawa, CAN; 2 Medicine, University of Calgary, Calgary, CAN; 3 Alberta Health Services, University of Calgary, Calgary, CAN; 4 Information Technology, University of Calgary, Calgary, CAN; 5 Emergency Medicine, University of Calgary, Calgary, CAN

**Keywords:** low back pain, choosing wisely, imaging, plain radiographs, emergency medicine

## Abstract

Objectives

The Choosing Wisely Canada (CWC) Emergency Medicine group recommends avoidance of lumbosacral radiographs for patients with non-traumatic low back pain (LBP) in the absence of red flags. The objective of this study was to evaluate imaging practices of emergency physicians (EPs) in four Calgary emergency departments (EDs) and identify patient, physician, and environmental factors associated with over-ordering of radiographs for low-risk LBP patients.

Methods

Data was retrospectively collected from patients, ages 18–50 and Canadian Triage and Acuity Scale (CTAS) codes 2–5, who presented with non-traumatic LBP to Calgary EDs from April 1, 2014 to March 31, 2016. Patients considered high risk, specifically with partial thromboplastin time (PTT) > 40 seconds or international normalized ratio (INR) > 1.2 seconds, any consult, admission to hospital, and history of cancer, were excluded. The primary outcome was to establish the overall usage of lumbosacral radiographs. The secondary outcome was to identify factors that influenced lumbosacral spine imaging.

Results

Data from 2128 low-risk patients showed that 14.8% of the patients received lumbosacral radiographs. Variation among 132 physicians in X-ray ordering ranged from 0% to 90.9%. There were site-specific differences in ordering patterns [Rockyview General Hospital (RGH) = 21.6% > South Health Campus (SHC) = 15.6% > Peter Lougheed Centre (PLC) = 13.1% > Foothills Medical Centre (FMC) = 9.7%, p < 0.001]. Canadian College of Family Physicians-Emergency Medicine (CCFP-EM) licensed physicians ordered more X-rays compared to Fellow of the Royal College of Physicians of Canada (FRCPC) licensed physicians (16.6% vs. 11.1%, p < 0.001). Older physicians and physicians with more experience ordered more X-rays than their younger and less experienced colleagues.

Conclusion

Considerable variation exists in the ordering practices of Calgary EPs. Overall, EPs seem to be choosing wisely in terms of ordering plain radiographs for non-traumatic LBP.

## Introduction

Choosing Wisely Canada (CWC) is a campaign to help patients and healthcare professionals engage in conversations about unnecessary tests and treatments [1]. On June 2, 2015, the Canadian Association of Emergency Physicians (CAEP) established a list of five CWC recommendations that included the following recommendation: “Don’t order lumbosacral (low back) spinal imaging in patients with non-traumatic low back pain (LBP) who have no red flags/pathologic indicators.” Since then, the CAEP has updated the list to include five more CWC recommendations for emergency department (ED) physicians [1, 2].

Low back pain is a problem that affects 80–85% of people at some point in their lives [3]. Back pain is one of the top 10 reasons for visit to emergency departments in Canada [4]. In 2011, more than 150,000 Canadian emergency department visits were with a chief complain of back pain [4]. The prevalence of emergency department visits due to LBP in 12 countries was estimated to be 4.4% [5].

It has been established that imaging for LBP in the absence of red flags (Table 1) is rarely helpful in the management of these patients [6-10]. In the absence of red flags, patient outcomes are no better in regards to pain, function, mental health and quality of life when imaging is performed [10]. On the contrary imaging of LBP causes patients to be exposed to unnecessary harmful ionizing radiation that could increase their likelihood of developing cancers, myeloproliferative disorders, and aplastic anemia in the future [6]. Unnecessary imaging may also lead to incidental findings and overdiagnosis of conditions that can trigger more unnecessary testing. It has been shown that patients are receiving more surgery for lower back issues due to unnecessary imaging [11, 12]. In addition, this unnecessary imaging causes increased wait times in EDs and financial burdens on the healthcare system [6, 13]. The purpose of this study was to assess the practices of ED physicians for the imaging of low-risk back pain in the four EDs in Calgary. Subsequently, we identified patient, physician, and environmental factors that influence the rates of imaging for low-risk back pain.

**Table 1:** Red flags of low back pain.

Red flags of low back pain
Age of onset > 50 years
History of cancer
Unexplained weight loss
Fevers, night sweats
Traumatic back pain
Saddle anesthesia
Acute onset of urinary retention or incontinence
Loss of anal tone or fecal incontinence
Weakness or loss of sensation in lower extremities
Intravenous drug use
Prolonged use of steroids
Immunosuppression

## Materials and methods

Data was retrospectively collected for patients who presented to one of the four Calgary EDs [Foothills Medical Centre (FMC), Peter Lougheed Centre (PLC), Rockyview General Hospital (RGH), South Health Campus (SHC)] with low-risk LBP over a two-year time frame, between April 1, 2014 and March 31, 2016. Low-risk patients were defined based on inclusion and exclusion criteria. Included in the study were patients who were between the ages of 18 and 50. Patients included in the study had non-traumatic back pain with an International Classification of Disease 10 (ICD-10) diagnosis code of M54.5. Furthermore, patients who received any consult in the ED or who were admitted to the hospital were excluded. Patients with an elevated partial thromboplastin time (PTT) or international normalized ratio (INR) or Canadian Triage and Acuity Scale (CTAS) 1 were excluded as well (Table 2). The data was collected using Sunrise Clinical Manager (SCM) and Sunrise Emergency Care (SEC), an electronic system used in all Calgary emergency departments to track patient flow and order entry data. The data was not collected by a formal chart review. The past medical history and triage notes were reviewed and intravenous (IV) drug users, patients with history of cancer or steroid use were excluded if that information was provided in the triage note. A total of 2128 patients and 132 physicians met eligibility criteria. Each physician at least had five back pain cases that met all inclusion criteria. However, the 132 physicians included had at least 10 low back pain patients. Patient factors analyzed in the study were patient age and gender. Physician factors analyzed were physician age, gender, years of practice and type of residency program [Canadian College of Family Physicians-Emergency Medicine certification (CCFP-EM) vs. Fellow of the Royal College of Physicians of Canada (FRCPC)]. CCFP-EM is a three-year residency program where residents are trained as family physicians for the first two years and then have one-year intensive training in the emergency medicine to obtain certification. On the other hand, FRCPC is a five-year residency training program after which physicians are licensed in emergency medicine. The environmental factors analyzed, with respect to each ED visit, were site (FMC vs. PLC vs. RGH vs. SHC), day of the week (weekday vs. weekend), time of the day (day vs. evening vs. night), time from triage to physician encounter, seven-day revisit to ED, seven-day admission to hospital after ED discharge, and the date of presentation to ED (pre-CWC recommendation vs. post-CWC recommendation). A pRoject Ethics Community Consensus Initiative (ARECCI) screening tool was used for ethics and we received a score of two, which deemed our project to be of minimal risk [14]. To further minimize the risk, patient and physician data were anonymized and stored in a password-protected file. Microsoft-Excel and Statistical Package for the Social Sciences (SPSS) programs were employed for chi-squared and t-test statistical analyses of the data.

**Table 2:** Inclusion and exclusion criteria. ED: Emergency department; ICD: International classification of disease; CTAS: Canadian triage and acuity scale; INR: International normalized ratio; PTT: Partial thromboplastin time; IV: Intravenous.

Inclusion Criteria	Exclusion Criteria
Patients > 18 years	INR > 1.2 seconds, PTT > 40 seconds
Patients ≤ 50 years	Active or previous cancer
Discharge home from ED	IV drug use, steroid use
Non-traumatic back pain (ICD-10 code M54.5)	Any consult or hospital admission
CTAS2-5	CTAS 1

## Results

Patient and physician demographic information

Among the 2128 patients included in the study, 1027 (48.3%) were female and 1101 (51.7%) were male. Patients 18–50 years of age were included in the study (Table 3). The mean age of patients in the study was 35.6 years (Standard deviation (SD) = 8.3). The mean age of patients in the study who did not receive a lumbar spine X-ray was 35.5 years (SD = 8.3), and the mean age of patients in the study who received a lumbar spine X-ray was 35.8 years (SD = 8.5). The average number of patients seen by all physicians included in the study was 16. Among the 132 physicians included in the study, 31 (23.5%) were female and 101 (76.5%) were male. Eighty-six (65.2%) out of the 132 physicians were CCFP-EM trained, and 46 (34.8%) were FRCPC trained (Table 3). Physicians aged 29.7 to 70.7 years were included in the study (Mean (M) = 43.9, SD = 9.7). Physicians with 0.5 to 44 years of practice were included in the study (M = 10.0, SD = 9.2).

**Table 3:** Patient and physician demographics. CCFP-EM: Canadian College of Family Physicians-Emergency Medicine; FRCPC: Fellow of the Royal College of Physicians of Canada; SD: Standard deviation.

		No. (%)	Range (mean, SD)
Patient gender	Female	1027 (48.3)	
	Male	1101 (51.7)	
Patient age			18-50 years (35.6, 8.3)
Physician gender	Female	31 (23.5%)	
	Male	101 (76.5%)	
Physician training program	CCFP-EM	86 (65.2)	
	FRCPC	46 (34.8)	
Physician age			29.0-70.7 years (43.9, 9.7)
Number of years in practice for physicians			0.5-44.0 years (9.4, 9.1)

**Figure 1:**
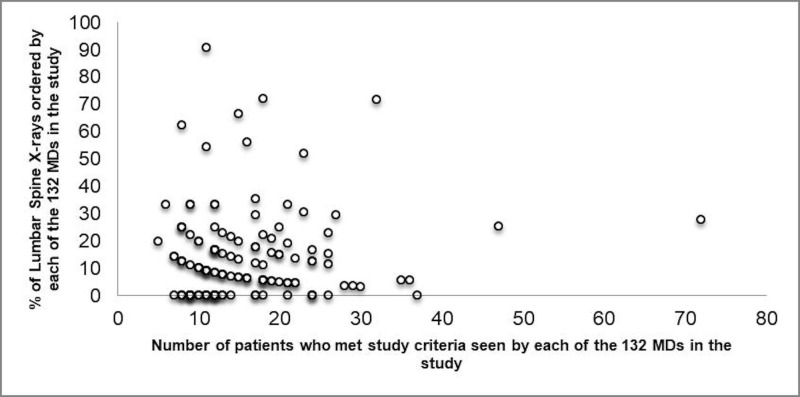
Figure depicting the variation in lumbar spine radiographs ordered with respect to the number of patients seen by each of the 132 MDs included in the study. MD: Doctor of Medicine.

Physicians ordered X-rays on 315 out of the 2128 patients, which amounts to 14.8% of the low-risk LBP patients included in the study. The variation in X-ray ordering was 0 to 90.9% with an interquartile range (IQR) of 0 to 20% (Figure 1).

Patient factors

There was no statistical difference in X-ray ordering based on patient age (t (2126) = -0.660, p = 0.509). Furthermore, patient gender did not influence the X-ray ordering rates of physicians (p = 0.273) (Tables 4, 5).

Physician factors

CCFP-EM trained physicians ordered more X-rays as compared to FRCPC trained physicians (16.6% vs. 11.1%, p < 0.001). The mean years of practice of physicians who did not order a lumbar spine X-ray was 8.9 years (SD = 8.5 years), and the mean years of practice of physicians who did order a lumbar spine X-ray was 12.1 years (SD = 11.6 years). Physicians with more years of experience ordered more X-rays than physicians with less years of experience (t (2126) = -5.809, p < 0.001). The mean age of physicians who did not order a lumbar spine X-ray was 43.0 years (SD = 9.3 years), and the mean age of physicians who did order a lumbar spine X-ray was 46.0 years (SD = 10.8 years). Older physicians ordered more X-rays than younger physicians (t (2126) = -5.047, p < 0.001). The data was not divided based on physician age, but the mean age of physicians who ordered lumbar spine X-ray (46 years) was significantly higher than the mean age of physicians who did not order lumbar spine X-rays (43 years). The rates of X-rays ordered did not depend on the gender of the physician in charge (Tables 4, 5).

Environmental factors

The site of presentation influenced the X-rays ordering rates of physicians (RGH = 21.6% > SHC = 15.6% > PLC = 13.1% > FMC = 9.7%, p < 0.001). All other environmental factors, including day of the week of presentation, time of day of presentation, triage to doctor of medicine (MD) time, seven-day ED revisit, seven-day hospital admission, and date of presentation with regards to pre- and post-CWC recommendation did not influence the X-ray ordering practices of physicians (Tables 4, 5).

**Table 4:** Results for nominal variables. ED: Emergency department; MD: Doctor of Medicine; CWC: Choosing Wisely Canada; FMC: Foothills Medical Centre; PLC: Peter Lougheed Centre; RGH: Rockyview General Hopsital; SHC: South Health Campus; CCFP-EM: Canadian College of Family Physicians-Emergency Medicine; FRCPC: Fellow of the Royal College of Physicians of Canada.

		Lumbar spine X-ray (n = 315)	No lumbar spine X-ray (n = 1813)	
		No. (%)		P Value
Patient gender	Male	154 (14.0)	947 (86.0)	0.273
	Female	161 (15.7)	866 (84.3)	
Day of week	Weekday	228 (15.4)	1252 (84.6)	0.237
	Weekend	87 (13.4)	561 (86.6)	
Time of day	Day (07:00-14:59)	147 (16.1)	768 (83.9)	0.211
	Evening (15:00-22:59)	103 (13.1)	685 (86.9)	
	Night (23:00-06:59)	65 (15.3)	360 (84.7)	
ED site	FMC	44 (9.6)	416 (90.4)	<0.001*
	PLC	83 (13.1)	551 (86.9)	
	RGH	97 (21.6)	352 (78.4)	
	SHC	91 (15.6)	494 (84.4)	
ED revisit rates in 7 days	No	304 (15.0)	1717 (85.0)	0.176
	Yes	11 (10.3)	96 (89.7)	
Admission rates in next 7 days	No	313 (14.8)	1802 (85.2)	0.953
	Yes	2 (15.4)	11 (84.6)	
MD gender	Male	254 (14.9)	1453 (85.1)	0.84
	Female	61 (14.5)	360 (85.5)	
MD program	CCFP-EM	237 (16.6)	1186 (83.3)	<0.001*
	FRCPC	78 (11.1)	627 (88.9)	
ED visit date: Pre and Post-CWC recommendation	Pre (April 1, 2014-June 2, 2015)	201 (15.6)	1083 (84.4)	0.172
	Post (June 3, 2015-March 31, 2016)	114 (13.6)	730 (86.5)	

**Table 5:** Results for continuous variables. SD: Standard deviation; MD: Doctor of Medicine.

	Lumbar spine X-ray ordered (n = 315), Mean (SD)	No lumbar spine X-ray ordered (n = 1813), Mean (SD)	T (2126)	P value
Patient age (years)	35.8 (8.5)	35.5 (8.3)	0.66	0.509
Physician years of practice	12.1 (11.6)	8.9 (8.5)	5.81	<0.001*
Physician age (years)	46.0 (10.8)	43.0 (9.3)	5.05	<0.001
Triage to MD time (hours)	2.1 (1.6)	2.1 (1.4)	0.61	0.545

## Discussion

The mean X-ray ordering rate for low-risk LBP patients in Calgary EDs was found to be 14.8%. Thirty-four out of the 132 physicians ordered X-rays on 0% of their low-risk LBP patients, whereas some ordered X-rays on a high percentage of their patients with the maximum being 90.9%. Even though there was significant variation between the X-ray ordering rates of Calgary ED physicians, on average it appears that Calgary ED physicians are choosing wisely in regards to ordering plain radiographs for low-risk low back pain. In comparison, a study performed in a Halifax emergency department from 2009 to 2015 showed that 27.4% of patients with LBP received plain radiographs [15]. This was similar to the rates of plain radiographs ordered for LBP in United States emergency departments (30.5%) as studied by the National Hospital Ambulatory Medical Care Survey (NHAMCS) from 2002 to 2006 [16]. In Australia, 25% of LBP patients received referrals for imaging by their general practitioners from 2001 to 2008 [17].

This study showed that there was no difference in the X-ray ordering rates of physicians before and after the CWC ED recommendation on LBP was published, specifically June 2, 2015 (15.6% vs. 13.6%, p = 0.172). This implies that there was not a change in practice by Calgary ED physicians after the CWC guidelines were published. There was either no significant change or a small decline in imaging of low-value back pain in the United States after their Choosing Wisely campaign was launched [18, 19]. In Australia, similar results were seen, as there was no significant difference in imaging practices of general practitioners before (23.9%) and after (25.3%) the National Health and Medical Research Council guideline was released [17].

The X-ray ordering rates by Calgary ED physicians were impacted by several physician factors. CCFP-EM trained physicians ordered X-rays more frequently than FRCPC trained physicians (16.6% vs. 11.1%, p < 0.001). Older physicians, as well as physicians with more years of experience, ordered more X-rays than younger and less experienced physicians. It has been suggested that there are many reasons for lack of adherence of physicians to practice guidelines, including lack of knowledge of guidelines, lack of belief in guidelines, fear of litigation, patient expectations, and financial incentives [20]. It has been observed previously that the type and timing of training has an impact on physician practices. It was surprising to find that older and more experienced physicians are ordering more X-rays than less experienced physicians. This might be due to the fact that more recent graduates are taught about resource stewardship and Choosing Wisely initiatives in their curriculum. As certain groups of physicians are more likely to order X-rays for low-risk patients, education efforts can be targeted to those groups of physicians who were found to more frequently order X-rays.

The hospital site that the patient presented to did impact the X-ray ordering rates of physicians (RGH = 21.6% > SHC = 15.6% > PLC = 13.1% > FMC = 9.7%, p < 0.001). The rate of the lumbar spine radiograph ordering varies significantly based on the site of presentation of the patient. This might be due to local practices and culture related to radiograph use. It can be inferred that RGH has a local culture of more liberal radiograph use. The lower rates in ordering spine radiographs at FMC might be due to FMC being a higher acuity hospital that relies more on advanced imaging.

All other environmental factors investigated did not impact the X-ray ordering practices of physicians. It was observed that the imaging practices of physicians were not influenced by the day of the week or the time of the day that the patient presented to the ED. The imaging practices were also not influenced by the wait times in ED. This is reassuring as this means that physician practices are consistent and not altered by external factors.

In order to study if there were consequences of not ordering lumbar spine X-rays for back pain, seven-day ED revisit and admission rates were examined. There was no difference whether or not a lumbar spine X-ray was ordered for both these variables. This further strengthens the conclusion that there is no need to order lumbar spine X-rays for low-risk back pain patients.

This study yielded a lot of important information regarding plain radiograph imaging practices of low risk low back pain by ED physicians, however, it does have limitations. This study was not a formal chart review, limiting the amount of patient information that was considered in the study. This also meant that our exclusion of patients was subjected to the charting inconsistency of physician descriptions of patient presentations. Furthermore, the definition of low risk was based on the information available on the electronic system. We were unable to exclude patients with constitutional symptoms, cauda equina, infection or neurological findings on physical exam. Also, the study was a retrospective cohort study that was confined to Calgary and therefore may lack applicability to other centers.

## Conclusions

Calgary ED physicians are choosing wisely in regards to imaging low-risk LBP patients. However, considerable variation exists between physicians with certain groups ordering X-rays more frequently than others. CCFP-EM trained physicians, physicians with more years of experience and older physicians are ordering X-rays more frequently than FRCPC trained physicians, physicians with less years of experience and younger physicians, respectively. The site of presentation of the patient also influenced the rate of X-rays ordered for low-risk patients. To the best of our knowledge, this was the first study to investigate factors that influence imaging practices of low risk low back pain by emergency physicians.
